# Quantifying the liquid–liquid transition in cold water/glycerol mixtures by intermolecular hyperfine relaxation-induced dipolar modulation enhancement (ih-RIDME)

**DOI:** 10.1039/d5cp03647j

**Published:** 2025-11-07

**Authors:** Sergei Kuzin, Maxim Yulikov

**Affiliations:** a Department of Chemistry and Applied Biosciences, ETH Zurich Zurich Switzerland myulikov@ethz.ch sergei.kuzin@mpinat.mpg.de

## Abstract

Water/glycerol mixtures are commonly used for experiments with biomacromolecules at cryogenic temperatures due to their vitrification properties. Above the glass transition temperature, they undergo liquid–liquid phase separation. Using a novel EPR technique called intermolecular hyperfine relaxation-induced dipolar modulation enhancement (ih-RIDME), we quantified the molar composition in frozen water/glycerol mixtures with one or the other component deuterated after the phase transition. Our experiments reveal a nearly equal phase composition regardless of the proton/deuterium isotope balance. With the new ih-RIDME data, we can also revisit the previously reported body of glass transition data for these mixtures and build a consistent picture of water/glycerol freezing and phase transitions. Our results also indicate that ih-RIDME has the potential to be used for investigating the solvation shells of spin-labelled macromolecules.

## Abstract 2

Pulse EPR allows to quantify the liquid–liquid transition in water/glycerol mixtures above glass transition temperature. This is achieved using Hahn echo decay and ih-RIDME experiments.

## Graphical abstract

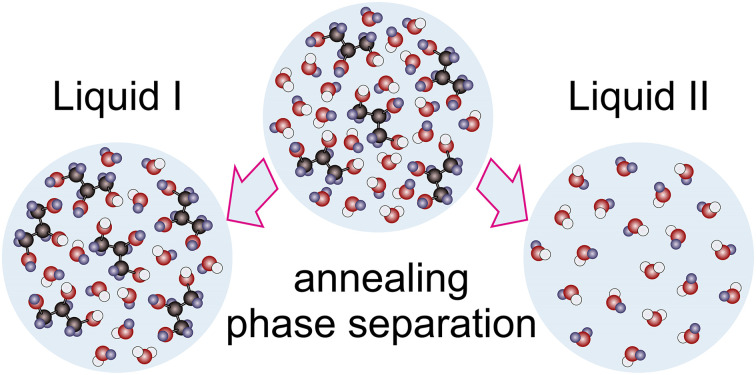

## 1 Introduction

For deeply cooled or frozen solvent mixtures, knowing the local phase state and local composition, as well as possibly inhomogeneous local density distributions for each individual molecular type, besides the fundamental interest, can also be essential for performing advanced spectroscopic experiments. In particular, this applies to magnetic resonance: different types of solid-state (SS) nuclear magnetic resonance (NMR),^1–4^ dynamic nuclear polarisation (DNP),^5,6^ or pulse electron paramagnetic resonance (EPR).^7,8^ Similar considerations might also appear to be important beyond magnetic resonance spectroscopy, in any other spectroscopy, crystallography or microscopy measurements at low temperatures, *e.g.* in the currently boosting field of cryo-electron microscopy.^9–11^

In magnetic resonance and beyond, solvents and solvent mixtures that can form glass upon shock-freezing are of practical importance. Ideally, the solvent mixture must form an amorphous bulk phase, without major solvent crystallisation. This prevents EPR- or NMR-active solutes from locally concentrating or even precipitating, thereby maintaining a uniform distribution of dissolved molecules. With this condition being fulfilled, EPR and NMR can be used to study the molecular organisation of the glass around the probes.

The structure, free energies and compositions of the solvation shells of various solutes at ambient temperature have been addressed in the last few years by THz calorimetry and by water dynamics measurements using Overhauser DNP.^12–17^ In particular, water dynamics in water/glycerol mixtures at ambient temperatures have been studied.^12^ Also, for such mixtures at ambient temperatures, contributions of bulk water, wrap water (around hydrophobic moieties) and bound water were evaluated, and the Gibbs free energy, the entropy and the enthalpy of mixing were determined.^14^ This set of data allows for a better verification of the MD computations' quality. Also, the comparison of Overhauser DNP, THz calorimetry and MD enabled the discussion of coordination motifs (tetrahedral, icosahedral) in water/glycerol mixtures.^12,14^ The solvation properties of biopolymers are fundamentally related to their interactions with other molecules, solubility, folding, liquid–liquid phase separation, gel or fibre formation, *etc.* However, is the molecular organisation of the glassy phase around large solutes like proteins, nucleic acids, and polysaccharides upon freezing close to that at ambient temperature? Normally, a positive answer is assumed as it allows for the transfer of low-temperature data to the native conformational state of macromolecules. On the other hand, glass is a metastable state; therefore, its local structure can change with time and affect the solute. It is attractive to address these questions using pulse EPR, as it offers a variety of powerful characterisation methods. Freezing samples for pulse EPR measurements changes the balance between entropic and enthalpic contributions to the chemical potential of mixing of each type of solute and solvent molecules. The corresponding changes in the solvent mixture composition might appear or might be, *e.g.* kinetically hindered. Following such changes would be useful for shedding light on the room temperature solvation properties.

Here, we investigated frozen water/glycerol mixtures, which are most commonly used for studying biomacromolecules at cryogenic temperatures. However, one of the standard storage conditions for protein and nucleic acid solutions is *T* = −80 °C (193 K). This is above the glass transition temperature, estimated at 158 ± 5 K by differential scanning calorimetry for a pure water/glycerol mixture with 50% (w/w) glycerol fraction^18^ and in the range of 164–190 K from EPR data with spin probes and spin-labelled protein molecules at *ca.* 50% volume fraction of glycerol.^7^ In general, considering any glass-forming solvent mixture, frozen samples stored above the glass transition temperature (*T*_g_) exist in the form of a supercooled liquid. This is a state of matter between the glass transition and melting temperatures, characterised by the absence of long-range order and high but finite viscosity. Under these conditions, the modification of the structure of the deeply cooled liquid may occur, some mechanical stress may relax, and a fraction of the solvent may partially crystallise or undergo other phase transitions. These processes are referred to as glass ageing.^19,20^ It was also found that above the glass formation temperature, deeply cooled water/glycerol mixtures can undergo isocompositional liquid–liquid transition (LLT) into glycerol-rich (Liquid I) and water-rich (Liquid II) phases at the length scale below 1 µm, without macroscopic phase separation.^21^ Such an LLT has been reported to be mainly driven by the local restructuring of water rather than glycerol.

In ref. 7, the local rearrangement of hydrogen bonds formed between solvent molecules and the spin labels has been observed in water/glycerol around the glass transition temperature. The recently introduced intermolecular hyperfine relaxation-induced dipolar modulation enhancement (ih-RIDME) technique^22–24^ is well-suited to probe the composition and inhomogeneities of solvent molecule distributions around paramagnetic centres, with the sensitivity region well beyond the first solvation shell, thus complementing the previously published hydrogen bonding data and revealing the overall composition of solvents in the spin probe's vicinity.

**Fig. 1:**
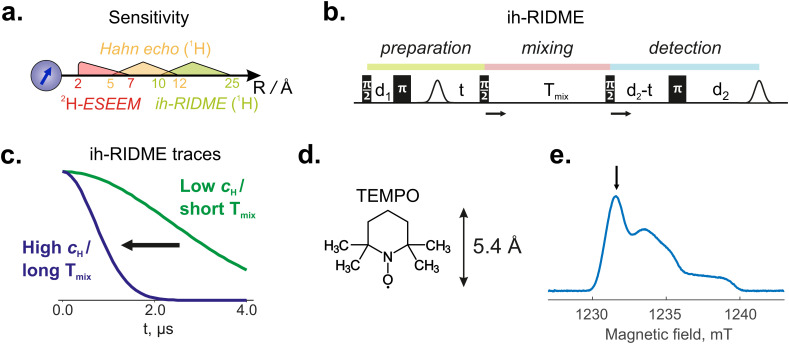
(a) Approximate sensitivity ranges of pulse EPR methods: ^2^H-ESEEM, Hahn echo decay and ih-RIDME. Numbers on the axis correspond to distances from the electron spin in Å. (b) The pulse sequence of five-pulse ih-RIDME with marked preparation, mixing and detection parts. Pulses are labelled based on their flip angles. The mixing block is progressively shifted, causing echo decay. (c) The decay of the ih-RIDME traces is steeper at higher proton concentrations or at longer mixing times. (d) Chemical structure of nitroxide radical TEMPO used in this work. All protons in this molecule are below the sensitivity region of ih-RIDME.^22^ (e) Echo-detected EPR spectrum of TEMPO in freshly frozen solution H_w_D_g_. The arrow indicates the positions of all EPR measurements.

In the present paper, we compare the ih-RIDME data for protonated and deuterated water/glycerol mixtures with the data from more conventional pulse EPR techniques, such as Hahn echo decay and matrix-peak electron spin echo envelope modulation (ESEEM). All three methods probe weakly coupled magnetic nuclei in solvents, albeit at different distances to the spin probe (see Fig. 1a). With these pulse EPR data, we can quantify the LLT and assign nitroxide spin probes to persist in the glycerol-rich phase. These experiments exemplify the capability of ih-RIDME for the quantitative determination of the distribution of solvent molecules in the surroundings of spin probes, which has been earlier evaluated to cover up to 3 nm distances from the spin probe,^22,24,25^ depending on the solvent deuteration level. This also allows us to propose the ih-RIDME technique as a pulse EPR experiment for investigating the solvation of biopolymers.

## 2 Theoretical background for ih-RIDME

In the general picture, ih-RIDME is a pulse EPR experiment that aims at quantification of the local concentration of magnetic nuclei within a 2- to 3-nm range from the electron spin. Specifically, it connects the decoherence rate of the electron spin in the EPR experiment with the spatial density of magnetic nuclei of the same isotope (here ^1^H). In homogeneous media, the latter can be related to the nuclear concentration. Importantly, careful analysis of the ih-RIDME data also allows the determination of whether the nuclear environments of all electron spins in the sample are similar, which is referred to as a homogeneous sample. So far, the method has been sufficiently tested and optimised for measuring local proton densities, which is essential for the present work.

In this section, we introduce the principles of the ih-RIDME experiment and the physical model behind the data analysis carried out in this study.

### 2.1 Pulse sequence

The pulse sequence for the five-pulse ih-RIDME experiment is shown in Fig. 1b.^25^ In contrast to the conventional RIDME technique,^26^ which uses the same pulse sequence but relies on the spontaneous spin flips in a pair of coupled electron spins, the ih-RIDME experiment is designed to probe the interaction of a single electron spin with a ‘cloud’ of protons around it. A favourable condition for the ih-RIDME is a low concentration of the spin probe (<100 µM) to avoid strong electron spin–spin contributions.^27^ Where dilution of electron spins is challenging, the suppression of the electron–electron contributions can be aided by selecting lower measurement temperatures so that the typical longitudinal relaxation times of spin probes substantially exceed the mixing times necessary for the ih-RIDME. This step may involve a compromise on sensitivity.

The pulse sequence of the ih-RIDME experiment consists of preparation, mixing and detection blocks. The preparation block at first creates a primary echo to avoid the dead time problem.^26^ The electron coherence, refocused at the point of the echo, continues evolving for time *t* under the sum hyperfine field exposed by close-by magnetic nuclei. The first π/2-pulse of the mixing block converts coherence into a polarisation. After the mixing time *T*_mix_, the second π/2-pulse of the mixing block turns the polarisation back to the transverse plane. This coherence is then refocused by the last π-pulse, forming the detected echo. In the ih-RIDME experiment, the echo intensity is measured as a function of delay t at several chosen mixing times *T*_mix_ (Fig. 1b and c). The dependence of the signal on both delays is a characteristic of the proton environment of the electron spin, and we review the physical and mathematical description of this behaviour in Section 2.2.

### 2.2 Formalism of hyperfine spectral diffusion

It is generally known that the simultaneous evolution of the electron-nuclear spin system under hyperfine and homonuclear dipolar coupling leads to the spin decoherence phenomenon in pulse EPR.^28–32^ In the context of ih-RIDME, we introduce a multi-nuclear hyperfine field 
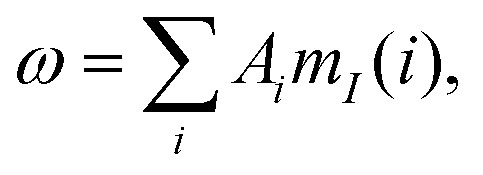
 where *A*_*i*_ are hyperfine couplings and *m*_*I*_(*i*) are nuclear spin projections (+1/2 or −1/2 for protons). The homonuclear interaction mixes the hyperfine levels, rendering the hyperfine field *ω* non-stationary after the electron spin is excited. In the ensemble, hyperfine field decorrelates during the time evolution of the electron spin, and this collective dynamics can be effectively described within the formalism of electron spin spectral diffusion, which underlies the analytical model for ih-RIDME.

First, we consider the state density function of multi-nuclear hyperfine levels, *ρ*(*ω*), called the hyperfine spectrum. For a realistic number of about 100 nuclei in the electron spin's vicinity, we deal with a high number 2^100^–10^30^ of closely spaced multi-nuclear states that form a quasi-continuous multi-spin hyperfine band. Thus, we can approximate *ρ*(*ω*) by a smooth zero-centred Gaussian function with standard deviation *σ*:1
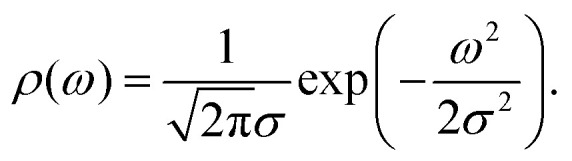
*σ* [freq] characterizes the entire nuclear bath around the electron spin. For locally homogeneous systems, where proton concentration is unambiguously defined, *σ* is proportional to it,^22^

2 *σ* ∝ *c*_H_

with the proportionality factor *σ*/*c*_H_ = 0.0215 MHz L mol^−1^.

In equilibrium, the EPR sample consists of spin packets with an individual hyperfine field *ω*. During the transverse evolution in the preparation block, such a packet gains the phase *ωt*. This is described by an ensemble function *μ*_*t*_(*ω*) = *ρ*(*ω*)exp(*iωt*) called the magnetization spectrum. During the mixing block, the hyperfine field fluctuates due to the homonuclear interaction. The effect of the homonuclear coupling can be simplified as continuous flip–flop-like oscillations in the closest spin pairs. As shown in the analytical solution with two nuclei, the effective oscillation frequency depends on both homonuclear coupling and the gradient of the hyperfine field within a nuclear pair.^31^ In amorphous matter with many protons, internuclear vectors are uniformly distributed over a sphere and, to a good approximation, uncorrelated from electron–nuclear vector orientation and length. Consequently, the frequencies of such nuclear pair nutations are broadly distributed. Also, due to the weak correlation of electron–nuclear and nuclear–nuclear interactions in the unstructured spin bath,^32^ the spectral diffusion kinetics allows a description by dissipative models, despite being formally a deterministic process. Mathematically, we describe this as a diffusion of the magnetization spectrum with the following equation:^22^3
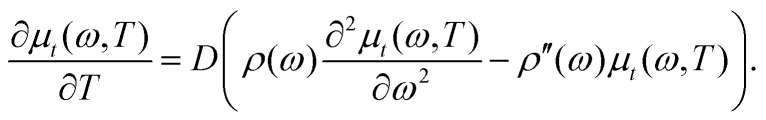
Here, *T* is the time after the beginning of the mixing block, and *D* is the spectral diffusion coefficient (up to a few hundred MHz^3^ µs^−1^). After integrating this equation of motion over the mixing block of length *T*_mix_ with initial condition *μ*_*t*_(*ω*, 0) := *ρ*(*ω*)exp(*iωt*), the ih-RIDME signal is calculated as follows:4
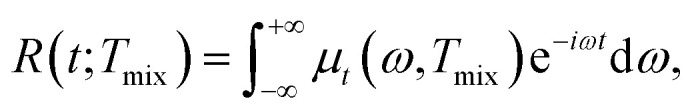
which encompasses the coherence evolution after the mixing block and the echo detection. Consequently, for the same electron spin, *ω* is different before and after the mixing block, and this is observed as the decay of echo intensity *R*(*t*) (Fig. 1c).

Comparing eqn (3) and (4), we emphasise that ih-RIDME traces are parametrised by two values: *D* and *σ*. If the solvent mixture is partially deuterated, retaining the chemical composition, both *σ* and *D* change. Experimentally, the relation*D* = *kσ*^3^was established, where *k* = *D*/*σ*^3^ [time^−1^] is sensitive to the topology of proton distribution.^22,24^ In particular, the parameter *k* correlates with an average number of close proton–proton contacts per single nucleus.^24^ In water/glycerol mixtures, the coefficient *k* is slightly different for water protons and for glycerol protons: *k* = 18.0 ± 1.2 µs^−1^ and *k* = 16.4 ± 0.8 µs^−1^, respectively.^22^ However, while the difference is detectable, it is so small that the ih-RIDME traces' shapes for a given mixing time accurately scale with the overall bulk proton concentration^22^, resulting in a shape match of the ih-RIDME traces for different proton concentrations after the time axis transformation *t* → *t*·*c*_H_, at all mixing times.^22^ Finally, in proton–deuterium mixtures, considered in the present work, deuterium-induced spectral diffusion is negligible.^22,24^

### 2.3 Sensitivity region in ih-RIDME

The nuclei that contribute to the ih-RIDME decay are those whose homonuclear interactions with neighbours are strong enough to mix the hyperfine levels. This condition typically does not hold for the nearest few nuclei in a strong gradient of the electron's magnetic field. Such nuclear spin pairs are then ‘frozen’ on the ih-RIDME time scale and do not contribute to the ih-RIDME signal decay. The electron–proton distance with maximum contribution to ih-RIDME, determined by equality of hyperfine coupling difference to the nuclear–nuclear coupling, amounts to *R*_sd_ ≈ 14 Å in fully protonated solutions.^22^ If the proton concentration is lower, the matching condition shifts to larger values as *R*_sd_ ∝ *c*_H_^−1/3^.^22^ Consequently, for half-deuterated solvents (see Section 3), the maximum sensitivity is expected at *R*_sd_ ≈ 17–18 Å. Upper boundary of the sensitivity window is not sharp and can be estimated to be 1.5–2*R*_sd_.^22^ Influence of protons at shorter distances is weaker and can be neglected at *R* < 0.5*R*_sd_. Hence, all protons of the TEMPO radical (Fig. 1d), used as a spin probe in this work, are not considered for the interpretation of the ih-RIDME data.

### 2.4 ih-RIDME data analysis

One can typically measure a series of decays with different mixing times and analyse them globally. The normalized data *V*(*t*; *T*_mix_) are described by a model^22,32^

5 *V*(*t*; *T*_mix_) = *R*(*t*; *T*_mix_)·*F*(*t*).

The *R*-factor is given by eqn (4) and the *F*-factor arises since transverse evolution under homonuclear coupling is not included in the derivation of the *R*-factor.

For the signal in the five-pulse ih-RIDME experiment, the previously proposed analytical forms were used in this work:^22,24^

6 *R*(*t*; *T*_mix_) ≈ exp(−*α*(*T*_mix_)*σ*^2^*t*^2^)

with *α*(*T*_mix_) = 1 − exp(−0.245*kT*_mix_), and

7 *F*(*t*) ≈ exp(−*βσ*^2^*t*^2^),

with *β* ≈ 0.13 for five-pulse ih-RIDME with delays [*d*_1_, *d*_2_] = [0.4, 4.2] µs. Eqn (6) was proposed *via* the numerical analysis of the exact solution (eqn (4))^24^, and eqn (7) was inferred from experimental data.^22^ Note that *β* is similar for both water and glycerol.^22^ However, it is different in different pulse sequences used for ih-RIDME and may slightly vary for different delays *d*_1_ and *d*_2_.^24^ From eqn (5)–(7), a Gaussian shape of the ih-RIDME traces is expected, whereby proton density *σ* determines the maximum coefficient in the exponent, (1 + *β*)*σ*^2^ at *T*_mix_ → +∞, and *k* regulates the build-up of this coefficient with mixing time (Fig. 1c).

We note that the ih-RIDME data can be analysed either directly using the numerical model (2–7) or by comparison with the reference data from a homogeneous sample of the known proton concentration. The comparison is done by matching the traces' shape *via* global scaling of the time axis (*i.e.* the same axis scaling for each trace in the series). The scaling coefficient is the ratio of the proton concentrations in two samples. Simultaneous analysis of multiple traces with varying mixing times ensures high accuracy in determining the proton concentration. If the ih-RIDME traces of the test sample cannot be precisely matched to those of the reference homogeneous sample, this is sufficient for concluding that the proton distribution around spin probes in the test sample is heterogeneous, *i.e.* the local proton concentration/density is different for different molecules of the spin probe.^23^ Consequently, in a biphasic sample, the absence of accurate ih-RIDME trace scaling serves as proof that the spin probes are present in both phases. On the other hand, if all spin probes are within only one of the two phases, the shape congruence of ih-RIDME traces must apply, and the local proton concentration can be determined. While the proton concentration in an unknown homogeneous sample can also be determined with certain accuracy by other pulse EPR methods, such as Hahn echo decay^30^ or ESEEM, the ih-RIDME technique is a tool to verify the homogeneity of an unknown sample in terms of the nuclear environment of spin probes.

## 3 Experimental section

### 3.1 Chemicals

D_2_O (Sigma Aldrich, 99.8 atom% D), 2,2,6,6-tetramethylpiperidine-1-oxyl (TEMPO) (Sigma Aldrich, 99%), H_8_-glycerol (C_3_H_5_(OH)_3_, Carl Roth, >99.7%), and D_8_-glycerol (C_3_D_5_(OD)_3_, Sigma Aldrich, >98 atom% D) were used without further purification.

### 3.2 Sample preparation

Stock solution of TEMPO in D_2_O (*c*(TEMPO) = 1 mM) was diluted 10 times with D_2_O or H_2_O and mixed with protonated or perdeuterated glycerol according to Table 1. All masses were controlled using an analytical balance. The mole fraction of glycerol in all solutions is in the range of 16–17%, corresponding to the volume fraction in the range of 44–45% and the mass fraction in the range of 47–51%. 30 µL of each solution was transferred into a quartz EPR tube with an outer diameter of 2.95–3.00 mm and shock-frozen in liquid nitrogen, ensuring the formation of a water/glycerol glass. The final concentration of TEMPO was 50 µM.

**Table 1:** List of the solvents used in this study, their proton–deuteron composition, and phase memory times (*T*_m_) of TEMPO in freshly frozen solutions, measured at 50 K. H and D correspond to protonated and perdeuterated chemicals. Proton fraction is calculated as 
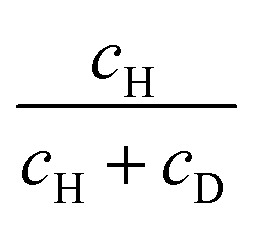
. A detailed description of solvent compositions can be found in SI (S1)

Sample	Water	Glycerol	*c*_H_, M	Proton fraction, %	*T*_m_, µs
H_w_H_g_	H	H	104	94.4	4.75 ± 0.02
H_w_D_g_	H	D	56.4	51.1	6.36 ± 0.02
D_w_H_g_	D	H	47.9	43.4	8.05 ± 0.02

The EPR samples were characterized immediately after preparation (‘fresh’), and then they were stored at −80 °C = 193 K (GFL Labor-Tiefkültruhen). This value is above 158 K, which is the *T*_g_ of the water/glycerol mixture.^18^ In order to test whether the spectroscopic changes from storing are reversible, we thawed and refroze the samples in EPR tubes. To this end, the tubes were turned upside down, and the sample volume was warmed up to room temperature while the quartz beyond the solution was kept in liquid nitrogen. This prevented the condensation of water vapours in the radical solution and the sliding of water droplets condensed above the solution upon storing it. The unfrozen solutions were kept in a liquid state for 5–10 seconds and shock-frozen in liquid nitrogen again.

### 3.3 EPR measurements

All pulse EPR measurements were performed using a Q-band Bruker ElexSys spectrometer (MW frequency: 34–35 GHz), equipped with a home-built resonator designed for oversized 3 mm tubes.^33^ Measurements were conducted at 50 K. Temperature stabilisation was established using an Oxford Instruments He-flow cryostat. The lengths of π/2- and π-pulses were *t*_π/2_ = 12 and *t*_π_ = 24, respectively. Hahn echo decay (π/2)–*t*–(π)–*t*–det (*t* is incremented) was measured with a starting delay *t* of 300 ns. The Hahn echo decay traces were used to determine the 1/*e* decay time (*T*_m_), as well as for analysing the ESEEM contribution from deuterium nuclei.

Five-pulse RIDME measurements: the pulse sequence is shown in Fig. 1b, time delays were set to be *d*_1_ = 0.4 µs and *d*_2_ = 4.2 µs. Values of mixing time were chosen as a geometric sequence *T*_mix_ = 15 × 2^*n*^ µs (*n* = 0, …, 5). In the RIDME measurements, the deuterium ESEEM-averaging protocol^34^ with 8 steps of 16 ns was used. All measurements were conducted at the maximum of the EPR spectrum (Fig. 1e).

## 4 Results and discussion

While the phase separation in water/glycerol mixtures^21^ has not yet been commonly appreciated in the magnetic resonance community, qualitatively the effect of ‘glass ageing’ is known to EPR spectroscopists: frozen solutions in water/glycerol stored at −80 °C transform from the transparent glassy state to an opaque, white state resembling snow. However, solutions shock-frozen directly to the liquid nitrogen temperature and handled far below −80 °C, *e.g.*, for EPR measurements at 50 K, or stored in liquid nitrogen, remain transparent. This appears to be associated with the phase separation in this cold mixture occurring above the glass transition temperature. Murata and Tanaka investigated LLT in a water/glycerol mixture and determined the stability boundary for the water-rich liquid II phase approximately in the temperature range of 150–200 K with the glycerol volume fraction changing between 30% and 50% as the temperature of the mixture decreases.^21^ Beyond this boundary, spontaneous phase separation into the liquid II and the glycerol-rich liquid I is followed by the crystallization of liquid II.

**Fig. 2:**
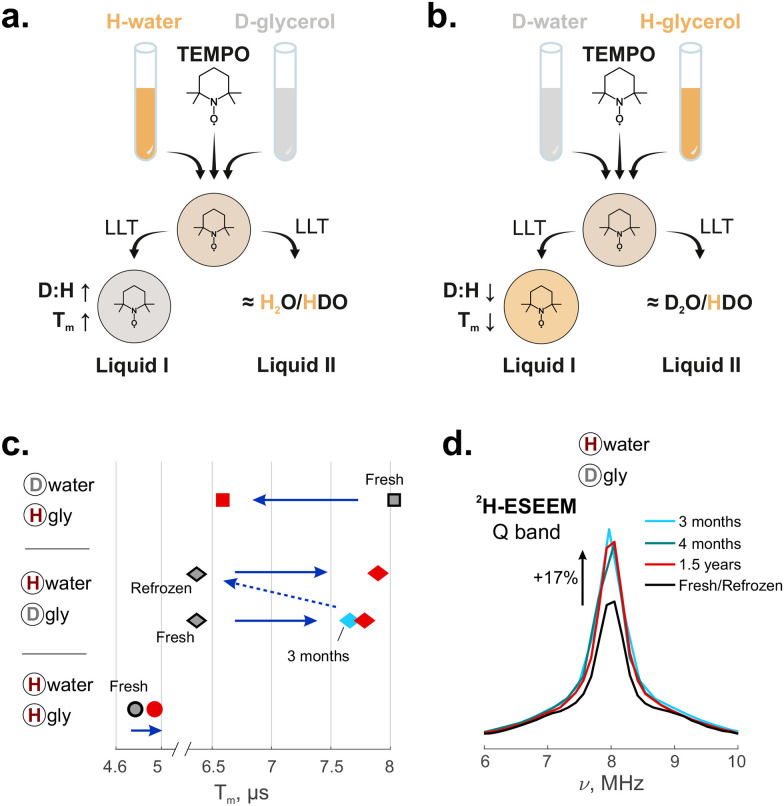
Spectroscopic changes of TEMPO in water/glycerol measured at the Q band, 50 K. (a) and (b) Schematic representation of the mixing and liquid–liquid transition (LLT) process for the cases of H_2_O and D_8_-glycerol (a) and D_2_O and H_8_-glycerol (b). The protonation degree of a solution is pictured as a shade of orange. According to the overall analysis, the TEMPO is selectively present in Liquid I, which explains the detection of changing local proton concentration with the total amount of protons in the sample being constant; (c) changes of decoherence times (times of 1/*e* decay of the Hahn echo): grey symbols correspond to freshly frozen solutions; red data were measured after storing the samples for more than 1 year in a −80 °C freezer. Each data point has an uncertainty of ±0.02 µs. Blue solid arrows indicate the direction of changes. A dashed arrow means refreezing the solution. The corresponding numeric values can be found in the SI (Table S2); (d) the two-pulse ESEEM spectra of H_w_D_g_ solution (^2^H matrix peak, normalized by the intensity of the overtone). The peak intensity increases after LLT, which indicates an increased deuterium concentration around TEMPO.

The idea of our study is to deuterate either glycerol (solution H_w_D_g_) or water (solution D_w_H_g_). After LLT in these mixtures, the new phases have a different water/glycerol composition; consequently, they exhibit a shift in the proton/deuterium ratio compared to the starting homogeneous mixture (Fig. 2a, and b). Changes in this ratio are probed by EPR, since decoherence times in Hahn-echo and ih-RIDME experiments are sensitive to the local proton environment of a spin probe. After numerous tests, we concluded that the observed EPR changes are fully reversible, meaning that refreezing the sample restores its transparency and resets the EPR properties to their initial values. Hence, one can exclude that the changes observed in aged samples are due to contamination by protonated water from the air, thereby verifying the interpretation of the EPR parameters changes as an internal transformation of the glass/cold-liquid structure.

### 4.1 Hahn echo decay and ^2^H-ESEEM at the Q band

For nitroxide radicals in electron spin-diluted solutions (below 100 µM) at cryogenic temperatures, the rate of Hahn echo decay, as a function of the interpulse delay *t*, is strongly affected by the local proton environment.^35–37^ It was shown that decay traces can be simulated with a static spin Hamiltonian that includes hyperfine and homonuclear dipolar interactions.^30,31^ At higher proton concentrations, the homonuclear interaction is stronger and the echo decays faster. The measured signal is the result of the deterministic evolution of the electron–nuclear spin system, where decay is due to the destructive interference of multiple nuclear modulation frequencies. For this reason, we prefer the term Hahn echo decay over transverse relaxation.

The effect of sample annealing at −80 °C on the Hahn echo decay (Fig. 2c) in the fully protonated medium is weak (4.0 ± 0.8% increase of the *T*_m_ time). In contrast, we observe substantial changes for the partially deuterated solvents: 22.5 ± 0.7% increase for H_2_O + D_8_-glycerol and 18.1 ± 0.5% decrease for D_2_O + H_8_-glycerol. The observed changes are reversible as refreezing of the solutions precisely restores the ‘fresh’ state. Within the ‘thaw–freeze–store’ cycles, the EPR changes are reproduced nearly quantitatively (SI, Section S2.3). For the shortest tested annealing time of 10 minutes, the LLT in the water/glycerol solutions was complete as judged from the Hahn echo data (SI, Fig. S2). Long storage at −80 °C up to approx. 1.5 years did not produce any further reliably detectable changes in the Hahn echo decay. As a reference, we stored a freshly frozen solution of H_w_D_g_ in liquid nitrogen for up to 5 weeks and did not detect any measurable modifications to the glass structure (see SI Section S2.1 for details).

The opposite direction of changes in the two partially deuterated mixtures indicates that the local rearrangement is primarily determined by the chemical nature of the mixture component – water or glycerol – rather than by the isotope composition. We can qualitatively explain all three observations by assuming that water is depleted from the solvent shell around the nitroxide during LLT (Fig. 2a and b). Glycerol has five non-exchangeable hydrogen atoms in its molecule. Therefore, a glycerol-enriched environment would cause a decreased local proton concentration with deuterated glycerol and an increased proton concentration with protonated glycerol. The transverse relaxation time should increase in the former case and decrease in the latter, as observed. Only minor changes are expected in this scenario when both solvents are protonated. The observed slight increase in the phase memory time in H_w_H_g_ solution cannot be attributed to the remaining D_2_O from the TEMPO stock solution (5%). The observed shift can be explained by differences in the shortest interproton distances in water and glycerol. In water, this is the intra-proton distance of 1.51 Å, and in glycerol, it is the distance within a CH_2_ group, 1.78 Å.^38^ Hence, typical homonuclear coupling is weaker in protonated glycerol than in protonated water, which reflects in the prolongation of *T*_m_.

Since H_w_D_g_ and D_w_H_g_ solutions are partially deuterated, we could also record the two-pulse ESEEM at the Q band, where the signal from deuterons has a sufficient modulation depth for the analysis and does not interfere with the proton signal (Fig. 2d). In all cases, only weakly coupled deuterons are seen around the Larmor frequency (*ν*_I_(^2^H) ≈ 8 MHz at the Q band). The stored samples are characterised by a different modulation depth compared to the fresh ones, namely, it increases in H_w_D_g_ solution by 16.6 ± 0.5% and decreases in D_w_H_g_ solution by 20.9 ± 0.7% (see SI Section S3). These results are consistent with the assumption that ageing depletes water in the vicinity of the nitroxide.

### 4.2 ih-RIDME analysis

The ih-RIDME data of fresh samples were published and analysed previously.^22^ We showed that it can be fitted well assuming a single value for *σ*. We interpret this by referring to fresh solutions as locally homogeneous. In principle, there is no long-range molecular order in glassy mixtures; therefore, the distribution of water and glycerol molecules within the sensitivity range of ih-RIDME can fluctuate. However, the sensitivity region appears large enough (as previously estimated, it is roughly a sphere of about 2 nm in radius for the given proton concentrations^22^). It contains many molecules, and thus large fluctuations in solvent composition have a negligible probability.

**Fig. 3:**
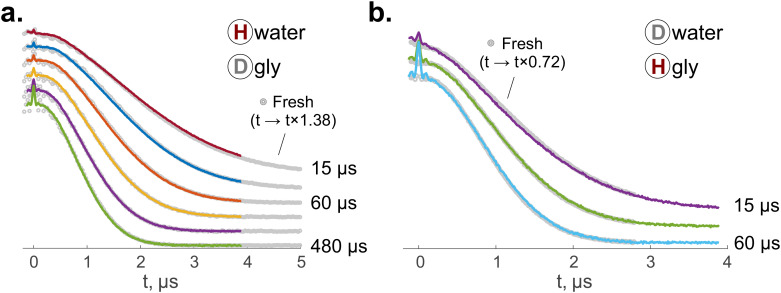
(a) and (b) Overlaying of the normalized ih-RIDME traces of TEMPO in fresh (grey dots) and stored samples (coloured dots) for the cases of (a) H_2_O and D_8_-glycerol and (b) D_2_O and H_8_-glycerol. The time axis of the fresh samples' data is scaled, and the scaling factors *f* are provided accordingly. The mixing times are specified next to the corresponding traces. The traces are shifted vertically for better visibility. Unscaled traces are available in the SI (Section S4).

Similar to the Hahn echo decays, the ih-RIDME traces of TEMPO in stored glasses deviate from those of the fresh samples. We found that the ih-RIDME data of the fresh and stored samples can be superimposed after stretching the time axis equally for the whole series of ih-RIDME traces measured at different mixing times. For the H_2_O + D_8_-glycerol sample, the RIDME decays of the fresh sample are faster compared to those of the stored sample. By applying a factor of 1.38 ± 0.01 to the ih-RIDME time axis of a fresh sample, we achieved the shape match for all traces simultaneously (Fig. 3a). The corresponding scaling factor for D_2_O + H_8_-glycerol is 0.72 ± 0.01 (Fig. 3b). The scaling values for H_w_D_g_ and D_w_H_g_ solutions are approximately inverse of each other, which stems from the near equality of the hydrogen atoms in water and glycerol. Hence, the ih-RIDME signal change is in agreement with the Hahn echo decay data. This can be expected, since both experiments rely on homonuclear coupling. However, the relative scaling in RIDME exceeds that of the relaxation data. This is evidence for the RIDME experiment's greater sensitivity to such changes in the local nuclear environment than the Hahn echo decay. The global scaling symmetry of the datasets, similar to the case of homogeneous solutions in ref. 22, indicates that the variations in the *k* = *D*/*σ*^3^ and *β* parameters are not significant for this work. This is expected as the types of protons in the mixture are the same before and after molecular rearrangements. The previously reported weak difference in *k* between water and glycerol protons^22^ is not resolved in these measurements. Consequently, the change in the ih-RIDME data results solely from the change of *σ*, which, in turn, can be quantitatively interpreted as a change of local proton concentration around TEMPO.

**Fig. 4:**
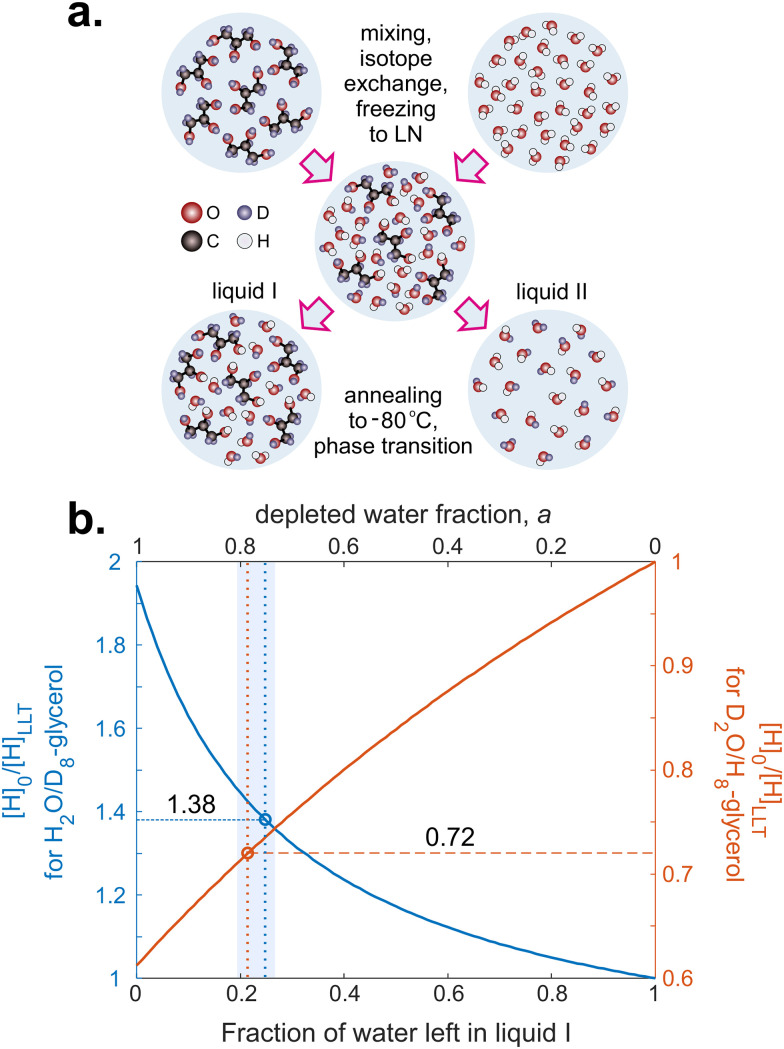
(a) Schematic molecular level representation of water and glycerol mixing, isotope exchange and liquid–liquid transition for the case of H_2_O and D_8_-glycerol. (b) Ratio of the initial proton concentration in the fresh sample ([H]_0_) and the proton concentration in liquid I after isotope exchange and liquid–liquid transition ([H]_LLT_) for the cases of H_2_O and D_8_-glycerol (blue curve, left vertical axis) and D_2_O and H_8_-glycerol (red curve, right vertical axis). Circles indicate the corresponding stretching factors for the ih-RIDME data, while the vertical band indicates the possible scatter of the molar fraction values due to measurement uncertainty and possible temperature variations during annealing.

To quantify the water/glycerol composition around TEMPO radicals after LLT, we derived the equations of proton balance, assuming the following points (illustrated in Fig. 4a): (i) chemical exchange of protons in water-based mixtures at room temperature is fast enough that it has fully equilibrated during the sample preparation;^39^ (ii) upon storing the sample above the glass transition temperature, water diffusion is active, and it may form ice due to the instability of the liquid II phase against crystallization;^21^ and (iii) water ice contains neither glycerol nor TEMPO molecules. In the balance equation, we operate with the local protonation degree,
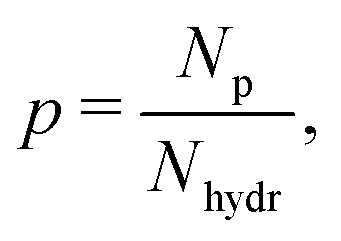
*i.e.* the ratio of protons (*N*_p_) to the total amount of protons and deuterons in the sensitivity region of ih-RIDME. The protonation degree before LLT (*p*_0_) is the same as the average solvent protonation degree calculated from the bulk isotope ratio. The total number of hydrogens in the mixture equals *N*_hydr_ = *h*_w_*N*_w,0_ + *h*_g_*N*_g,0_, where *h*_w_ = 2 and *h*_g_ = 8 are the number of hydrogen atoms in water and glycerol, respectively, and *N*_w,0_ and *N*_g,0_ are the initial number water and glycerol molecules in the mixture before LLT. In our model, we assume that water molecules diffuse in the cold, viscous mixture to form liquid II, followed by spontaneous crystallisation. Due to the hydrogen exchange with the hydroxyl groups of glycerol, the mean isotope composition of water is modified. For example, in H_w_D_g_ solution, OD deuterons of glycerol mix with OH protons of water, resulting in partial deuteration of the latter. We account for this by introducing the local protonation degree of water *p*_w_. In equilibrium, this number is equal to the protonation degree of chemically exchangeable protons8
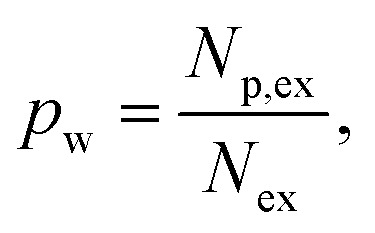
where *N*_p,ex_ is the number of exchangeable protons and *N*_ex_ is the number of all exchangeable hydrogens (protons and deuterons together). The latter is computed identically for both mixtures as *h*_w,ex_*N*_w,0_ + *h*_g,ex_*N*_g,0_ where *h*_w,ex_ = 2 and *h*_g,ex_ = 3 are the numbers of exchangeable protons in water and glycerol, respectively. In H_w_D_g_ solution, protons originate from water; therefore, they are all chemically exchangeable and *N*_p,ex_ = *N*_p_ = *p*_0_*N*_hydr_. This leads to the following formula for *p*_w_ in H_w_D_g_ solution9
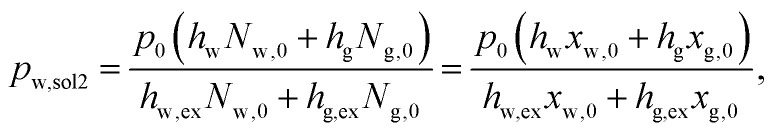
where we introduced *x*_w,0_ and *x*_g,0_ – molar fractions of water and glycerol in the mixture. For D_w_H_g_ solution, where protons stem from glycerol, we correct for non-exchangeable protons as *N*_p,ex_ = *N*_p_ − (*h*_g_ − *h*_g,ex_)*N*_g,0_, hence obtain10
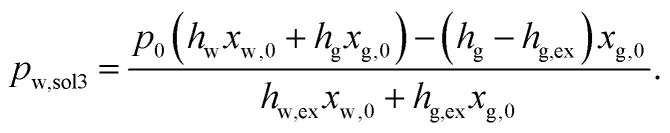


We describe the effect of LLT by a parameter 0 ≤ *a* ≤ 1, the relative fraction of water molecules that leave the solvent shell. In these terms, the observed local protonation degree after water depletion is11
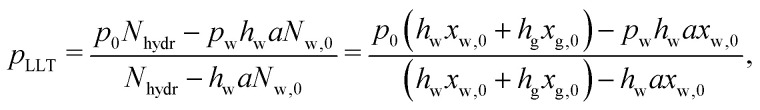
where *aN*_w,0_ is the average number of removed water molecules. Also, in this expression, we assumed that water depletion is an isotope-non-selective process. The ratio *p*_0_/*p*_LLT_, calculated for H_w_D_g_ and D_w_H_g_ solutions, is plotted as a function of *a* in Fig. 4b. For H_w_D_g_ solution, this curve is above 1, indicating that LLT always decreases the local proton concentration. For D_w_H_g_ solution, the situation is opposite, so the curve lies below 1.

With the direct proportionality relation between *σ* and *c*_H_ in ih-RIDME (see eqn (2)), we calculated *p*_LLT_ = *p*_0_/1.38 for H_w_D_g_ solution. Furthermore, eqn (11) was solved for *a* which yielded *a* = 75.1 ± 0.8%. For D_w_H_g_ solution, for which *p*_LLT_ = *p*_0_/0.72, we obtained *a* = 78.6 ± 2.1% (see Table 2). A more detailed composition of the water/glycerol environment probed by ih-RIDME is summarised in Table 3. These values are close for both solutions, albeit not the same, which may indicate the instability of the temperature regime at long incubations. At the same time, this might also be due to glass ageing being weakly sensitive to the isotope distribution between water and glycerol. Overall, one can conclude that the deuteration creates an efficient contrast for spectral diffusion with minimal perturbation of the LLT processes.

**Table 2:** Numerical details on the water depletion analysis in H_w_D_g_ and D_w_H_g_ solutions. *x*_0_ is the bulk water molar fraction, *p*_0_ is the bulk protonation degree, *p*_w_ is the water protonation degree after isotope exchange with glycerol, *f* is the ih-RIDME time axis stretching factor (see Fig. 3a and b) after LLT, *p*_LLT_ is the local protonation degree probed by TEMPO after LLT, and *a* is the calculated water depletion degree from liquid I. Uncertainties of *p*_LLT_ and *a* were propagated from the uncertainty of *f*

Solution	*x*_0_	*p*_0_	*p*_w_	*f*	*p*_LLT_	*a*, %
H_w_D_g_	0.840 ± 0.001	0.511 ± 0.001	0.700 ± 0.001	1.38 ± 0.01	0.370 ± 0.002	75.1 ± 0.8
D_w_H_g_	0.839 ± 0.001	0.434 ± 0.001	0.223 ± 0.001	0.72 ± 0.01	0.603 ± 0.008	78.6 ± 2.1

**Table 3:** Comparison of the water/glycerol composition around the spin probe before and after LLT based on the ih-RIDME data. *x* and *ϕ* represent molar and volume fractions of water *w* and glycerol *g*, respectively. All values are expressed as percentages

Solution	*x*_w_	*x*_g_	*ϕ*_w_	*ϕ*_g_
Before LLT				
H_w_D_g_	84.0 ± 0.1	16.0 ∓ 0.1	56.5 ± 0.1	43.5 ∓ 0.1
D_w_H_g_	83.9 ± 0.1	16.1 ∓ 0.1	56.3 ± 0.1	43.7 ∓ 0.1

After LLT				
H_w_D_g_	56.7 ± 0.8	43.3 ∓ 0.8	24.4 ± 0.6	75.6 ∓ 0.6
D_w_H_g_	52.3 ± 1.2	47.7 ∓ 1.2	21.6 ± 1.6	78.4 ∓ 1.6

### 4.3 Discussion

All three EPR methods provide a consistent picture of the proton/deuterium ratio shift in the vicinity of TEMPO upon LLT. The ^2^H-ESEEM method at the Q band is a short-range characterization method. It efficiently probes deuterium in the first solvation shell of TEMPO, and then its sensitivity decreases to *R*^−6^ for an individual deuterium nucleus.^40^ For *T*_m_, the sensitivity range of 5–12 Å has been proposed.^41^ For ih-RIDME, as discussed in Section 2.3, the approximate sensitivity range is 10–25 Å. Thus, the three methods complement each other (see Fig. 1a).

We can quantitatively compare the data from Hahn echo decay and ih-RIDME experiments. In ref. 30, Hahn echo decay for perdeuterated TEMPO in water/glycerol mixtures was measured in a full deuteration range, and the relation *T*_m_ ∝ *c*_H_^−0.65^ was proposed. We recalculated relative changes in *T*_m_ into changes in proton concentration using this power law and found good agreement with ih-RIDME. After LLT, *c*_H_ increases by a factor of 1.36 in H_w_D_g_ solution (1.38 from ih-RIDME) and 0.74 (*i.e.*, decreases) in D_w_H_g_ solution (0.72 from ih-RIDME). We note that exact shape congruence for Hahn echo decays is not expected because (i) the TEMPO methyl tunneling contribution^42^ does not scale with bulk proton concentration and (ii) the stretched exponential parameter *ξ* also changes with proton concentration.^30^ Consequently, a quantitative analysis of the proton bath contribution in Hahn echo decays requires the explicit fitting of these contributions with model functions.

ih-RIDME reports the state of the solvent only within 2–3 nm from the spin label (see Section 2.3). Thus, in the solution with TEMPO concentration of 50 µM, the observed volume is less than 0.1%. How can we infer the non-local properties from this? First of all, we can safely conclude from the ih-RIDME data that the majority of the spin probes are in the glycerol-rich liquid I phase. This follows from the precise shape congruence of the ih-RIDME traces. Should spin probes populate both phases and be present in two very different environments in terms of proton concentration, global matching by traces scaling would not be possible. Secondly, we know that fresh samples are locally homogeneous; therefore, liquid I must also be homogeneous. This also follows from the shape congruence, meaning that the liquid I has the same proton concentration throughout the sample volume. Besides, the composition of liquid I must correspond to thermodynamic equilibrium at −80 °C, which follows from reproducibility of changes of Hahn echo and ih-RIDME traces upon LLT (see data with solution H_w_D_g_ in Fig. 2c).

In principle, local cluster formation is possible in such solvent mixtures, with examples for water/glycerol and water/ethanol being reported.^30,43^ The sensitivity range for ih-RIDME, however, substantially exceeds the characteristic size range of such clusters, smearing out the effect of possible cluster formation. Again, this directly follows from the accurate shape congruence of the ih-RIDME traces.

At a TEMPO concentration of 50 µM with the uniform spin distribution, the average inter-probe distance is 32 nm. Ref. 21 evidences that in frozen water/glycerol mixtures, the changes during the phase separation processes take place on a micrometre scale. However, EPR data suggest that TEMPO molecules are far from the water agglomeration regions. It is thus likely that the TEMPO species are pushed from the nucleation and crystallisation centres together with the 2–3 glycerol-rich solvent sphere. This may be related to the high viscosity of glycerol in the liquid state. It forms a branched network of strong hydrogen bonds around TEMPO molecules, keeping them caged in the rigid frame. Alternatively, we can assume that the viscosity of the cold water/glycerol mixture at the storage temperature still allows TEMPO molecules to diffuse and distribute uniformly in the glycerol-rich phase.^44^

Based on the new ih-RIDME data, and the previously reported hydrogen bonding data for nitroxide radicals in water/glycerol mixtures, we can now draw the overall ageing picture of frozen TEMPO solutions at different length scales. Two main types of transformation appear. The first one, at the nearest vicinity to the spin centre, is the breaking and building of H-bonds with the NO-group of the nitroxide radical. This has been efficiently probed previously *via* ESEEM and electron–nuclear double resonance (ENDOR) spectroscopies.^7^ Such a pathway is the most sensitive to solvent deuteration due to the isotope effect on the hydrogen bond strength. Interestingly, direct contact of TEMPO with water molecules and formation of a new hydrogen bond were demonstrated upon annealing. This appears counterintuitive at first glance since, in fact, our ih-RIDME and other EPR data demonstrate that water depletion takes place in the broader vicinity of TEMPO molecules upon annealing, which is the second type of transformation. We can argue that the molecular reorganisation and LLT at low temperature are due to a shift in the balance between enthalpic and entropic contributions to the solvent and solute chemical potentials. It is likely that the small energy differences in the specific interaction between water and glycerol molecules favour their separation at lower temperatures, whereas they cannot do so at ambient temperature due to the stronger entropic terms.

Murata and Tanaka reported the formation of only small water crystals, approximately 11 nm in size, after LLT in water/glycerol mixtures below 205 K, whereas above this temperature, macroscopic ice growth has been observed.^21^ Since the incubation temperature in our experiments is only eight to ten degrees below this limit, and also our incubation conditions admit some temperature variations in the range of a few Kelvin, we cannot exclude bulk ice formation in our stored/annealed samples. From the fraction of water leaving the vicinity of spin probes (75%), we conclude that about 42% of the volume is occupied by the water-rich phase after the LLT. Thus, under our experimental conditions, it must turn to water crystals, leaving the remaining 58% of the sample volume in a deeply cooled liquid I state.

The separation of the water/glycerol mixtures is a bulk effect and is therefore most likely solute-independent, at least at the given µM-range of solute (TEMPO) concentrations. The overall vibrational modes distribution in the new glass formed after ageing should not be very different from the original one in the homogeneous water/glycerol mixture, because we observe only a weak change in the longitudinal relaxation time *T*_1_ of TEMPO radicals. An interesting experiment to disentangle different contributions would be a comparison of the glass evolution kinetics from EPR data, IR and terahertz spectroscopy, and phase-sensitive microscopy to correlate these changes across different lengths and time scales. To provide sufficient time resolution, such experiments would need to be conducted at lower temperatures, closer to the glass transition temperatures of the homogeneous mixture.

Having quantified the molar composition of the liquid I phase after LLT, we can also explain the apparent mismatch between the glass transition temperature for annealed and non-annealed samples.^7,18^ In the continuous wave (CW) EPR power saturation measurements for annealed and not annealed water/glycerol mixtures, a shift of the glass transition temperature was detected, which is in line with the LLT described by Murata and Tanaka and quantified here. Additionally, the time scale of the CW EPR measurements is quite long (tens of minutes to hours), which likely resulted in LLT occurring during the measurements above the glass transition temperature. The LLT is also the most realistic explanation for the increase in the saturation parameter *P*_1/2_ just above the glass transition temperature, resulting in a discontinuity in the saturation curve. The measurements on annealed samples indicated a higher glass transition temperature compared to the freshly frozen samples, consistent with the data for mixtures with a higher glycerol molar fraction appearing after LLT. Note that *T*_g_ of about 181 K after annealing is consistent with the liquid I molar composition reported in the present work. Thus, we can conclude that an accurate analysis of the LLT in water/glycerol mixtures and the phase quantification by ih-RIDME can also explain the seemingly inconsistent data in previously published reports.

Another question that appears interesting in this connection is the isotopic composition of the water-ice and amorphous water phases formed in the frozen mixture H_2_O–D_2_O, *e.g.* in the agarose-gel-stabilised samples without glycerol.^8^ On the one hand, the isotope effect on the strength of hydrogen bonds is well known. On the other hand, it is not well known whether this effect is strong enough to enable partial isotope separation upon fast freezing in supercooled isopentane, used in the cited work. As described above, in our RIDME-based experiments with water/glycerol mixtures, we obtained slightly different (relative difference by about 5%) compositions of the bulk phase around TEMPO radicals in the case of H-glycerol and D-water as compared to the reverted case of D-glycerol and H-water. While this might be within experimental uncertainty, *e.g.*, due to variations in temperature during annealing, it also leaves open the possibility of a weak isotope effect being present in these experiments. We can thus imagine that, due to the isotope effect, the process of ice formation, occurring at low temperatures and on a sufficiently slow time scale, might turn out to be partially isotope-selective. It would be interesting to verify or disprove this assumption in future experiments. It is possible that EPR experiments with more hydrophilic spin probes (*e.g.* 4-hydroxy-TEMPO), which eventually could remain in water-rich liquid II, would be informative.

The first solvation shell of TEMPO molecules, addressable in ENDOR and ESEEM experiments, mostly falls into the insensitive short distance range in ih-RIDME. However, having demonstrated here the usefulness of the ih-RIDME technique for determining the local compositions in deeply frozen water/glycerol mixtures, we can propose that ih-RIDME experiments on spin-labelled biomacromolecules might also appear useful for investigating the solvation shell peculiarities of protein residues in the vicinity of the spin-labelled site. In our lab, we have already demonstrated the applicability of ih-RIDME for studying polysaccharides^23^, and we are currently performing ih-RIDME tests on site-specifically spin-labelled protein molecules.

## 5. Conclusions

In this work, we demonstrated how pulse EPR can be utilised to investigate the liquid–liquid transition in cold water/glycerol mixtures. Thanks to the specific deuteration of water or glycerol, the local proton concentration in the vicinity of the TEMPO radical changes upon LLT, as probed by Hahn-echo and ih-RIDME experiments. The ih-RIDME traces show global shape congruence at all mixing times, allowing us to conclude that, firstly, TEMPO is located in the glycerol-rich liquid I, and secondly, the proton distribution in liquid I is homogeneous. By quantifying the local proton concentration using ih-RIDME, we calculated the volume ratio of liquid I to liquid II to be 52% : 48%. It is equivalent to say that upon LLT, 75.1 ± 0.8% (in H_2_O/D_8_-glycerol) and 78.6 ± 2.1% (in D_2_O/H_8_-glycerol) of water form a separate phase and crystallise.

New data help bring together the previously published results on glass transition temperatures, hydrogen bonding of spin probes and phase stability of water/glycerol solutions. The ih-RIDME technique proves to be a reliable and precise tool for determining the local molar composition in binary mixtures. It can therefore be proposed also for the tempting studies on the solvation of biomacromolecules.

## Author contributions

Both authors: conceptualization, methodology, investigation, formal analysis, writing – original draft, and writing – review and editing.

## Conflicts of interest

There are no conflicts to declare.

## Abbreviations

EPR: Electron paramagnetic resonance
NMR: Nuclear magnetic resonance
DNP: Dynamic nuclear polarization
ih-RIDME: Intermolecular hyperfine relaxation-induced dipolar modulation enhancement
ESEEM: Electron-spin echo envelope modulation
ENDOR: Electron–nuclear double resonance
LLT: Liquid–liquid transition
TEMPO: 2,2,6,6-Tetramethylpyperidine-1-oxyl

## Supplementary Material

CP-027-D5CP03647J-s001

CP-027-D5CP03647J-s002

## Data Availability

The data supporting this article have been included as part of the supplementary information (SI). Supplementary information is available. See DOI: https://doi.org/10.1039/d5cp03647j.
