# Supplementary material for: Quantifying the liquid–liquid transition in cold water/glycerol mixtures by intermolecular hyperfine relaxation-induced dipolar modulation enhancement (ih-RIDME)
Source: Phys Chem Chem Phys. 2025 Nov 7;27(48):25888–99. doi: 10.1039/d5cp03647j (PMC12641492; doi:10.1039/d5cp03647j)
Supplement: CP-027-D5CP03647J-s002 [file CP-027-D5CP03647J-s002.pdf]

Supplementary Information (SI) for Physical Chemistry Chemical Physics.
This journal is © the Owner Societies 2025

Supplementary Material: Quantifying the liquid-liquid transition in cold water/glycerol mixtures by ih-RIDME

Sergei Kuzin[1*], Maxim Yulikov[1*]

[1] Department of Chemistry and Applied Biosciences, Vladimir-Prelog-Weg 2, 8093, Zurich, Switzerland

*E-mail: sergei.kuzin@mpinat.mpg.de†, myulikov@ethz.ch

Contents

S1 Detailed mixture composition **S2**

S2 Hahn echo decay data **S3**

S2.1 Storing in liquid nitrogen . . . S3
S2.2 Annealing in dry ice . . . S3
S2.3 Storing at −80 °C . . . S4

S3 Two-pulse ESEEM **S6**

S4 ih-RIDME traces **S7**

†Current affiliation: Max Planck Institute for Multidisciplinary Sciences, Am Fassberg 11, 37077 Göttingen, Germany.

S1 Detailed mixture composition

	D_2O	H_2O	water	d_8-gly	h_8-gly	glycerol
**Mass fraction**						
$\mathbf{H}_w\mathbf{H}_g$	5.5	43.7	49.2	0	50.8	50.8
$\mathbf{H}_w\mathbf{D}_g$	5.4	43.5	48.9	51.1	0	51.1
$\mathbf{D}_w\mathbf{H}_g$	53.1	0	53.1	0	46.9	46.9
**Volume fraction**						
	D_2O	H_2O	water	d_8-gly	h_8-gly	glycerol
$\mathbf{H}_w\mathbf{H}_g$	5.6	49.2	54.7	0	45.3	45.3
$\mathbf{H}_w\mathbf{D}_g$	5.7	50.8	56.5	43.5	0	43.5
$\mathbf{D}_w\mathbf{H}_g$	56.3	0	56.3	0	43.7	43.7
**Molar fraction**						
	D_2O	H_2O	water	d_8-gly	h_8-gly	glycerol
$\mathbf{H}_w\mathbf{H}_g$	8.4	74.6	83.1	0	16.9	16.9
$\mathbf{H}_w\mathbf{D}_g$	8.5	75.5	84.0	16.0	0	16.0
$\mathbf{D}_w\mathbf{H}_g$	83.9	0	83.9	0	16.1	16.1

Table S1: Detailed description of water/glycerol mixture composition represented in mass, volume and molar fractions. Columns 'water' and 'glycerol' contain the sum values of all water and glycerol forms, respectively. All values are expressed as percentages.

S2 Hahn echo decay data

S2.1 Storing in liquid nitrogen

We demonstrated that storing water/glycerol mixtures deeply below the glass transition temperature preserves their EPR properties. Figure S1 shows Hahn echo decay traces $[\pi/2 - t - \pi - t - \text{echo}]$ of TEMPO in freshly frozen solution $\mathbf{H}_w\mathbf{D}_g$ (H_2O/d_8-glycerol, red line) and after storing it in liquid nitrogen (77 K) for 1, 3 and 5 weeks. Traces perfectly overlap, which demonstrates the stability of the water/glycerol glass under these conditions.

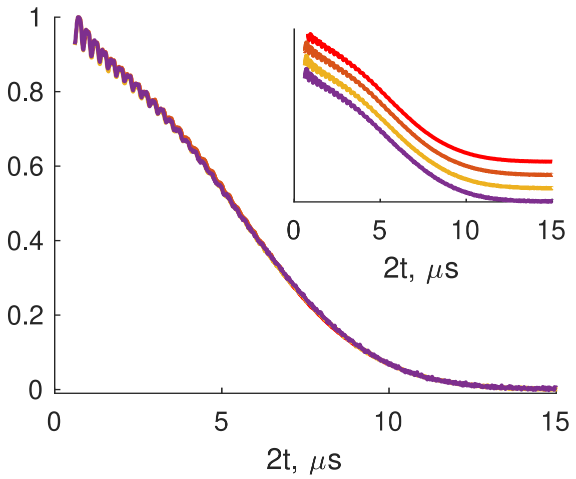

Figure S1: Superimposed and normalized Hahn echo decay traces of TEMPO in solution $\mathbf{H}_w\mathbf{D}_g$ stored in liquid nitrogen. The inset shows the same trace vertically shifted for better visibility. Storing conditions from top to bottom: freshly frozen, 1 week, 3 weeks, 5 weeks. Measurements were performed at Q band, 50 K.

S2.2 Annealing in dry ice

An experiment with short annealing in dry ice was performed as follows. An EPR tube with TEMPO in solution $\mathbf{H}_w\mathbf{D}_g$ (H_2O/d_8-glycerol) was freshly frozen in liquid nitrogen, and the Hahn echo decay was measured (Q band, 50 K - blue line in Figure S2). The decay time measured at $1/e$ of initial intensity amounted to 6.36 µs, which reproduces other measurements with fresh samples. After that, the tube was quickly immersed in the crushed dry ice. At this point, the sample in it was transparent. The top of the tube was firmly covered with Teflon tape to prevent water vapour condensation. After ten minutes, the tube, in which the content turned white and non-transparent, was rapidly transferred to liquid nitrogen for quenching further changes. The Hahn echo decay measurement was repeated (orange line in Figure S2), and the characteristic decay time increased to 7.93 µs. This value aligns with data obtained in other annealing experiments, which evidences that the LLT processes have completed.

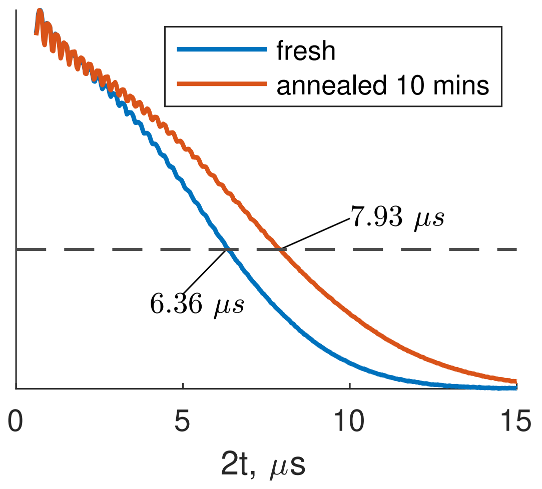

Figure S2: Normalised Hahn echo decays of TEMPO in solution $\mathbf{H}_w\mathbf{D}_g$ (H_2O/d_8-glycerol) as freshly frozen (blue) and annealed in dry ice for 10 mins (orange). The dashed line shows $1/e$ value. Measurements were performed at Q band at a temperature of 50 K.

S2.3 Storing at −80 °C

In Table S2, we collect values of T_m shown in Figure 2c in the main text. The corresponding time traces are shown in Figure S3.

Solution	State	T_m, µs
$\mathbf{H}_w\mathbf{H}_g$	Fresh	4.75
	Stored (> 1 year)	4.94
$\mathbf{H}_w\mathbf{D}_g$	Fresh	6.35
	Stored (> 1 year)	7.78
	Refrozen	6.37
	Stored (3 months)	7.67
	Stored (4 months)	7.78
	Refrozen	6.39
	Stored (1 week)	7.82
$\mathbf{D}_w\mathbf{H}_g$	Fresh	8.05
	Stored (> 1 year)	6.59

Table S2: Characteristic Hahn echo decay times for TEMPO in solutions $\mathbf{H}_w\mathbf{H}_g$, $\mathbf{H}_w\mathbf{D}_g$ and $\mathbf{D}_w\mathbf{H}_g$. 'Stored' refers to storing the EPR tube with solution at -80 °C in a commercial freezer. Data were measured at Q band at 50 K. The same uncertainty of ±0.02 µs applies to all values.

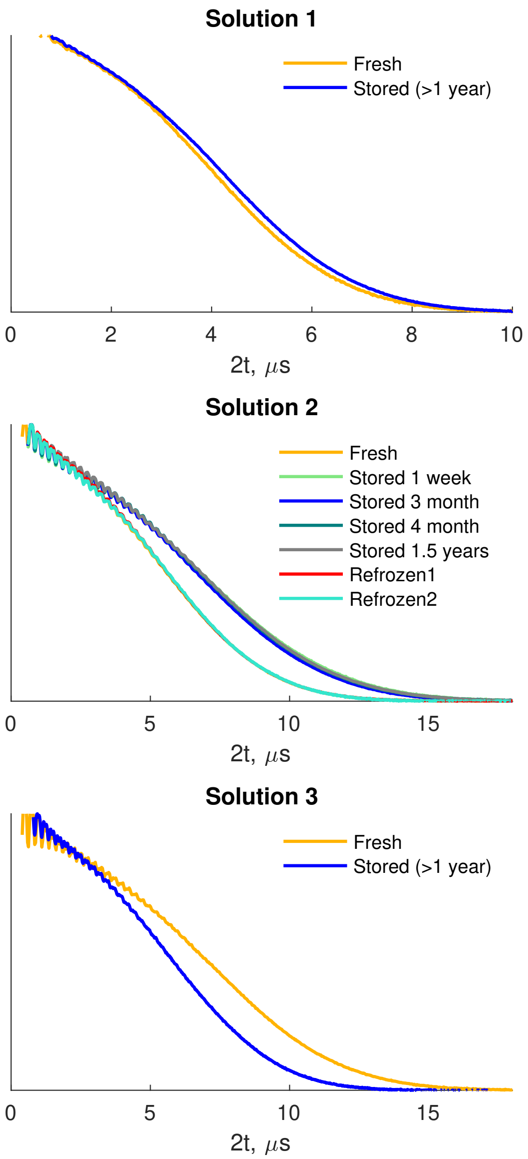

Figure S3: Normalised Hahn echo decay traces of TEMPO in solutions $\mathbf{H}_w\mathbf{H}_g$, $\mathbf{H}_w\mathbf{D}_g$ and $\mathbf{D}_w\mathbf{H}_g$: freshly frozen, stored at -80 °C and refrozen (see main text for details). The traces correspond to the values in Table S2.

S3 Two-pulse ESEEM

Figure S4 contains background-corrected two-pulse ESEEM traces of TEMPO in solutions $\mathbf{H}_w\mathbf{D}_g$ (H_2O/d_8-glycerol) and $\mathbf{D}_w\mathbf{H}_g$ (D_2O/h_8-glycerol) in the fresh state and after long incubation at −80 °C. Distinct oscillations with a period of ≈ 128 ns are observed, representing weakly coupled deuterium at Q band. Primary data were normalised by the maximum point; therefore, the amplitude of oscillations corresponds to the ESEEM modulation depth. For weakly coupled nuclei, the following approximation for the total modulation depth applies

$$k_{\text{tot}} \approx \sum_i k_i \qquad \text{(S3.1)}$$

where k_i is the single-nucleus ESEEM modulation depth. Consequently, k_{tot} can be a measure of the deuterium number in the vicinity of a spin probe. We observe opposite changes in solutions $\mathbf{H}_w\mathbf{D}_g$ and $\mathbf{D}_w\mathbf{H}_g$: after the liquid-liquid transition (LLT), the modulation amplitude in solution $\mathbf{H}_w\mathbf{D}_g$ increases, while in solution $\mathbf{D}_w\mathbf{H}_g$, it decreases. We interpret this as the parallel changes of local deuterium concentration. These data are compatible with the assumption that TEMPO is located in liquid I after the LLT (see Figure 2a-b in the main text).

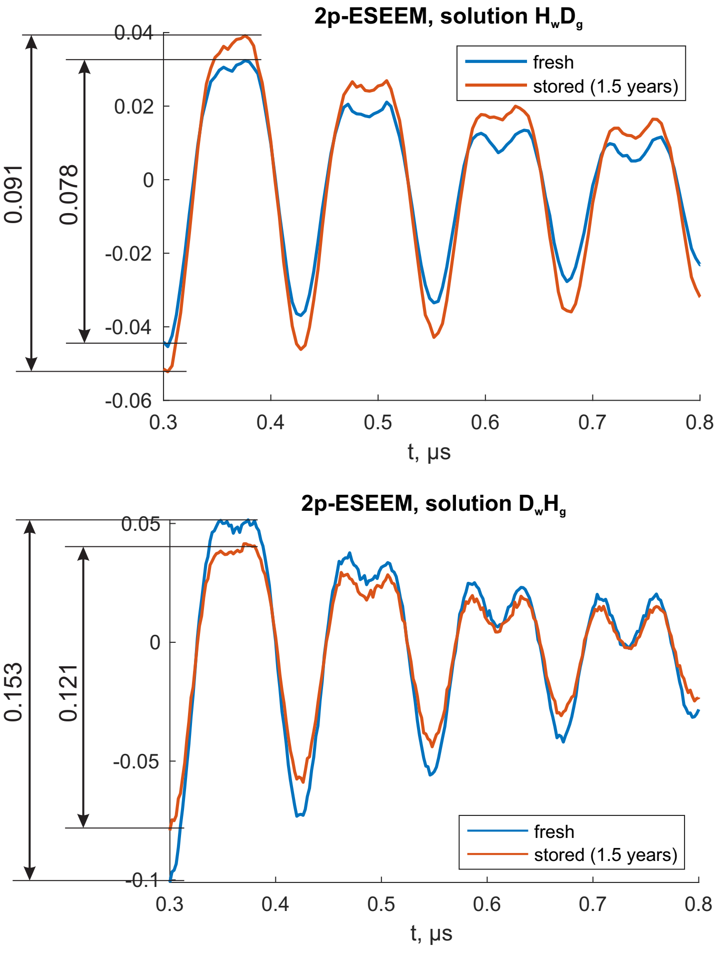

Figure S4: Fragments of two-pulse ESEEM traces for TEMPO in solution $\mathbf{H}_w\mathbf{D}_g$ (top) and solution $\mathbf{D}_w\mathbf{H}_g$ (bottom) as freshly frozen (blue line) and after long incubation at −80 °C (orange line). Traces were normalised by the maximum point and with the following subtraction of a polynomial background of order 4. Data are acquired at Q band, 50 K, as $\pi/2 - t - \pi - t -$ echo with initial delay $t = 0.3$ μs and step 4 ns.

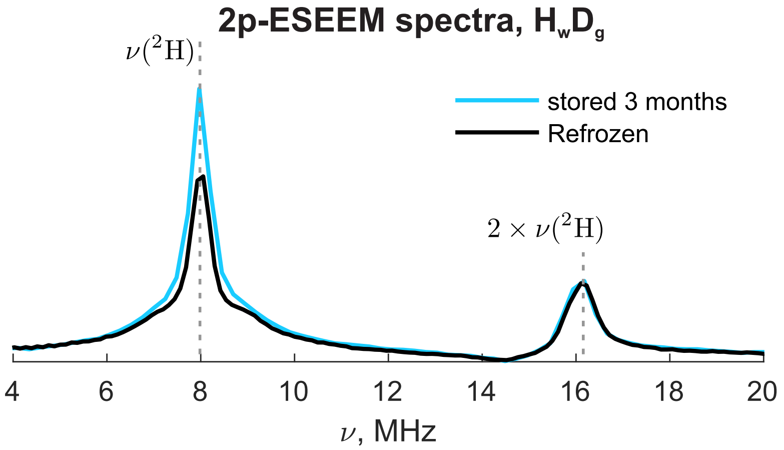

Figure S5: Two-pulse ESEEM spectra of fresh (black line) and stored (light blue line) solution $\mathbf{H_wD_g}$. The frequency range includes the matrix peak at the deuterium Larmor frequency and the combination peak. The spectra are normalised by the intensity of the combination peak.

S4 ih-RIDME traces

Figure S6 shows ih-RIDME traces of TEMPO in solution $\mathbf{H_wD_g}$ (H_2O/d_8-glycerol, left panel) and solution $\mathbf{D_wH_g}$ (D_2O/h_8-glycerol, right panel). Grey dots correspond to fresh samples and coloured lines represent samples after LLT (induced by storing at -80 °C). In solution $\mathbf{H_wD_g}$, the decays are slower after LLT, and in solution $\mathbf{D_wH_g}$, they are faster. This evidence is compatible with the assumption that TEMPO is located in liquid I after the LLT. The datasets for solution $\mathbf{H_wD_g}$ can be matched for all mixing times after multiplying the time axis of the fresh state by a factor of 1.38. The same applies to solution $\mathbf{D_wH_g}$, where the stretching factor amounts to 0.72 (see Figure 3a-b in the main text).

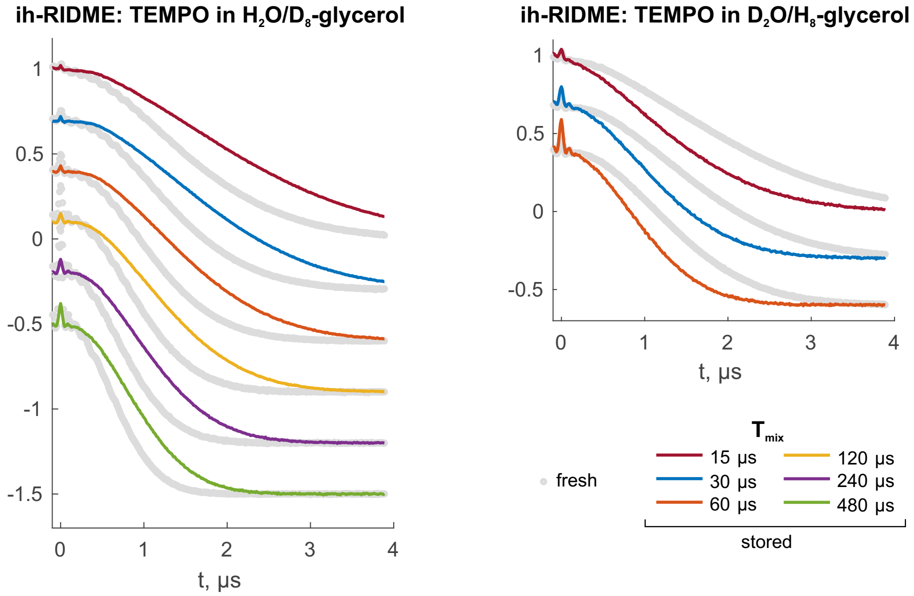

Figure S6: Experimental ih-RIDME traces of TEMPO in solution $\mathbf{H_wD_g}$ (right) and $\mathbf{D_wH_g}$ (left). Grey traces are measured with freshly frozen solutions. Coloured traces correspond to the stored samples. Traces are shifted vertically for better visibility. The legend with mixing times in the bottom right corner applies to both charts.
